# Highlights of Transesophageal Echocardiography During Interventions for Adult Congenital Heart Disease

**DOI:** 10.3390/jcm13226995

**Published:** 2024-11-20

**Authors:** Eihab Ghantous, Gentian Lluri

**Affiliations:** Ahmanson/UCLA Adult Congenital Heart Disease Center, Los Angeles, CA 90095, USA; glluri@mednet.ucla.edu

**Keywords:** adult congenital heart disease, transesophageal echocardiography, transcatheter procedure, imaging

## Abstract

Significant advances in the diagnosis and treatment of congenital heart disease have transformed patient outcomes, leading to an expanding adult congenital heart disease population. Many of these adults require lifelong procedural interventions, frequently performed in catheterization labs under the guidance of echocardiography. This review explores the transesophageal echocardiographic aspect in key catheterization-based procedures.

## 1. Introduction

Advancements in the diagnosis, medical therapies, and transcatheter and surgical interventions for congenital heart disease have led to decreased mortality and an improved quality of life for millions of patients [1,2]. Increased survival rates for congenital heart disease have resulted in the population of adults with congenital heart disease (ACHD) vastly surpassing that of pediatric patients [3,4]. These patients often require multiple procedures during their lives, many of which can be performed in catheterization labs using transcatheter methods. These procedures require knowledge of the patient’s history, thorough preprocedural planning with appropriate imaging techniques, and in most cases, the guidance of an experienced echocardiographer. This review aims to discuss the role of the echocardiographer, focusing on transesophageal echocardiography aspects of key catheterization-based procedures performed for adult patients with congenital heart disease.

## 2. Atrial Septal Defect (ASD) and Patent Foramen Ovale (PFO) Closure

**Figure 1:**
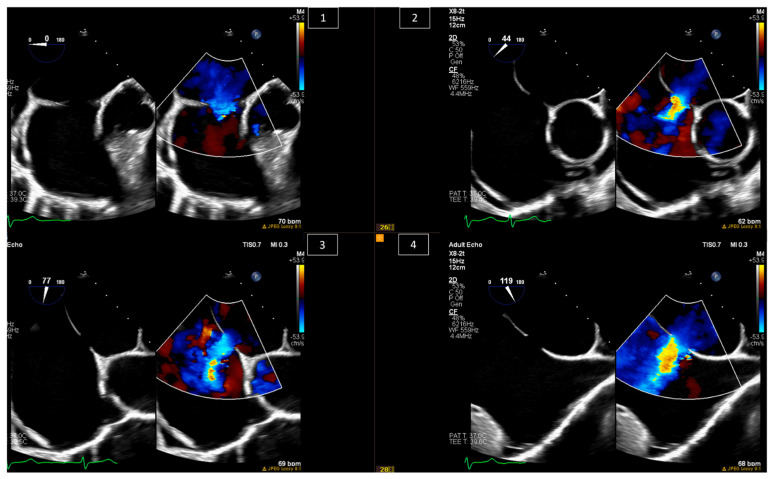
Baseline TEE images of a 43-year-old female with ostium secundum ASD closure during the percutaneous ostium secundum ASD closure procedure. Looking at the defect with the deficient rims at different angles, all with left-to-right shunt shown by color Doppler imaging: (**1**) At 0 degrees—atrioventricular valve rim with posterior rim. (**2**) At 40–60 degrees—retro-aortic rim with posterior rim. (**3**) At 70–90 degrees—superior rim and posterior/inferior vena cava rim. (**4**) At 90–130 degrees—superior vena cava rim and posterior/inferior vena cava rim.

**Figure 2:**
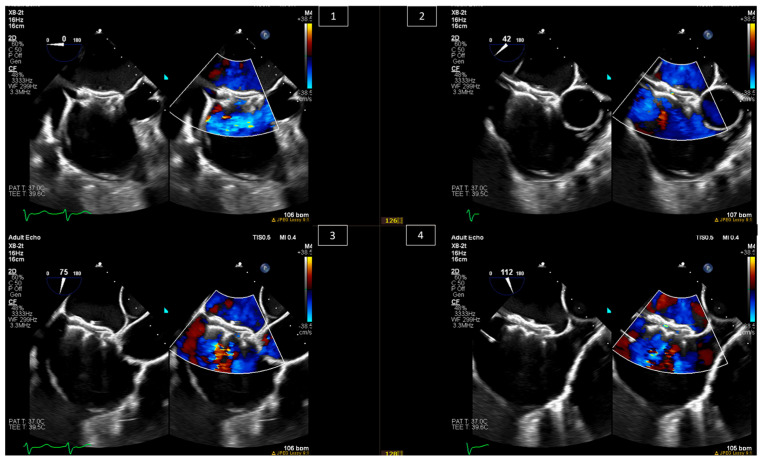
The same patient from Figure 1 during the same procedure, after deployment of the ASD closure device, confirming the position of the device in the different positions and its interaction with nearby structures and confirming no residual leak by color Doppler. (**1**) At 0 degrees—interaction with the aortic valve and mitral valve (not seen in the image). (**2**) At 40–60 degrees—interaction with the tricuspid valve and the aortic valve. (**3**) At 70–90 degrees—interaction with the superior vena cava and inferior vena cava. (**4**) At 90–130 degrees—again, looking at the interaction with the SVC and IVC from different angles.

Among congenital heart diseases, ASDs are considered one of the most common defects, accounting for about 10–15% of them [5,6]. They are divided into three major types based on anatomy: ostium secundum ASD, ostium primum ASD, and sinus venosus ASD [7]. Indications for interventions in ASD patients are thoroughly discussed in the American Heart Association/American College of Cardiology (AHA/ACC) and European Society of Cardiology (ESC) guidelines on the management of adult congenital heart disease [8,9], and these include symptoms, and in the absence of symptoms, significant left-to-right shunting (pulmonary blood flow–systemic blood flow ratio, or Qp:Qs > 1.5:1), paradoxical embolism, and/or the presence of right-heart enlargement. Percutaneous interventions are amenable to ostium secundum ASDs and sinus venosus ASDs, with over 80% of ostium secundum ASDs considered amenable to device closure [10]. Imaging during percutaneous ostium secundum ASD closure can be performed using intracardiac echo (ICE) only [11,12], although many centers still use transesophageal echocardiography (TEE). Imaging should be used periprocedurally to localize the shunt, understand the direction of the flow and the consequences of it, and look for other pathologies in the septum. During the procedure, imaging should be used to confirm the absence of clots in the left atrium, guide the positioning of the closure device, including balloon sizing and stop flow inflation, and confirm stability after deployment without injury to adjacent valves or myocardium and without new or increased pericardial effusion (Figure 1 and Figure 2).

**Figure 3:**
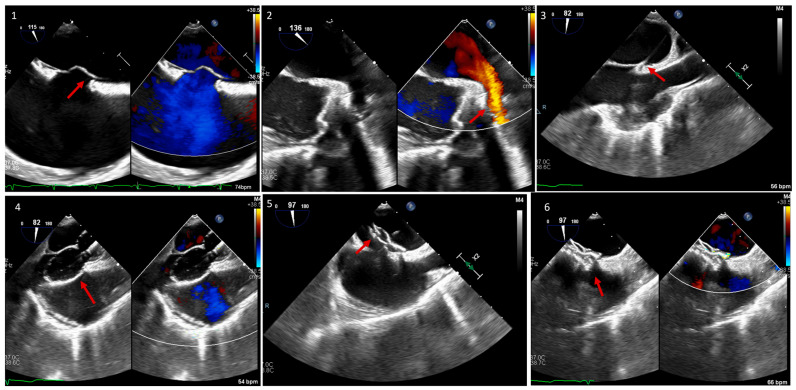
Baseline and interventional imaging during PFO closure of a 62-year-old male with a history of embolic stroke. (**1**) Looking at the interatrial septum with color Doppler and showing the PFO (arrow). (**2**) Looking at the pulmonary veins ensuring no anomalous pulmonary veinous return. In the image, the left lower pulmonary vein is seen at 136 degrees with flow towards the left atrium (arrow) with no anomalous connection. (**3**) Guiding the procedure—showing the wire crossing through the PFO (arrow). (**4**) Balloon inflation in the PFO and color Doppler confirming no other defects in the septum. (**5**) Deployment of the left atrial disc, confirming stable position and no interaction with the left atrial appendage and the aortic/mitral valve. (**6**) Deployment of the right atrial disc, confirming its stability, and using color Doppler to confirm no other shunts or defects in the interatrial septum.

For PFO closure, similar principles of peri-procedural imaging were applied (Figure 3).

**Figure 4:**
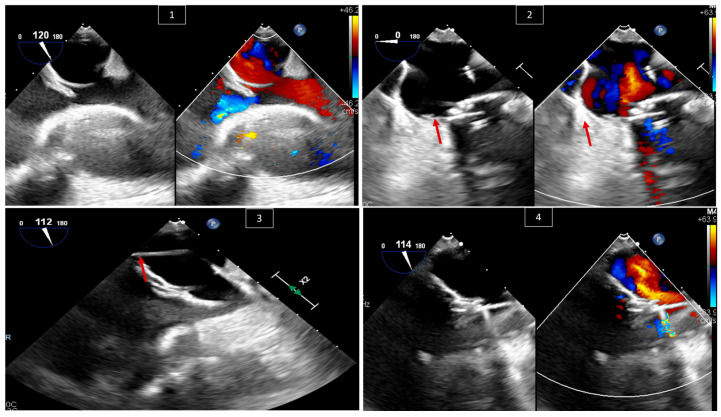
Guiding the percutaneous closure of the SSVD of a 70-year-old female. (**1**) The SVC right atrium (RA) and the sinus venosus defect seen with and without color Doppler with right upper anomalous pulmonary vein (RUPV) return in red at 120 degrees. (**2**) The same defect at 0 degrees with the wire (arrow) in the SVC and confirming its position in the RA and not through the defect. (**3**) Confirming the position of the wire (arrow) from the interatrial septum and in the right upper pulmonary vein (the anomalous vein) to check pressures during the intervention. (**4**) Post implantation of the covered stent in the SVC showing closure of the SSVD with no obstruction to pulmonary vein flow (in red).

Percutaneous superior sinus venosus defect (SSVD) closure was first described in 2014 by Garg et al. [13], having originally been presented during a conference in 2013 by Abdullah et al. [14]. Since then, the technique has gained considerable interest, and was adopted and modified by others to obtain a better result. A covered stent is deployed in the superior vena cava (SVC) in a way that closes the posterior wall defect, thereby closing the SSVD and redirecting anomalous pulmonary veins into the left atrium behind the covered stent [15]. As such, besides the routine screening of patients with ASD closure, TEE plays a crucial rule in diagnosing anomalous pulmonary veins and assures non-compression of them by balloon occlusion testing in the SVC and later by the covered stent (Figure 4).

## 3. Ventricular Septal Defect (VSD) Closure

**Figure 5:**
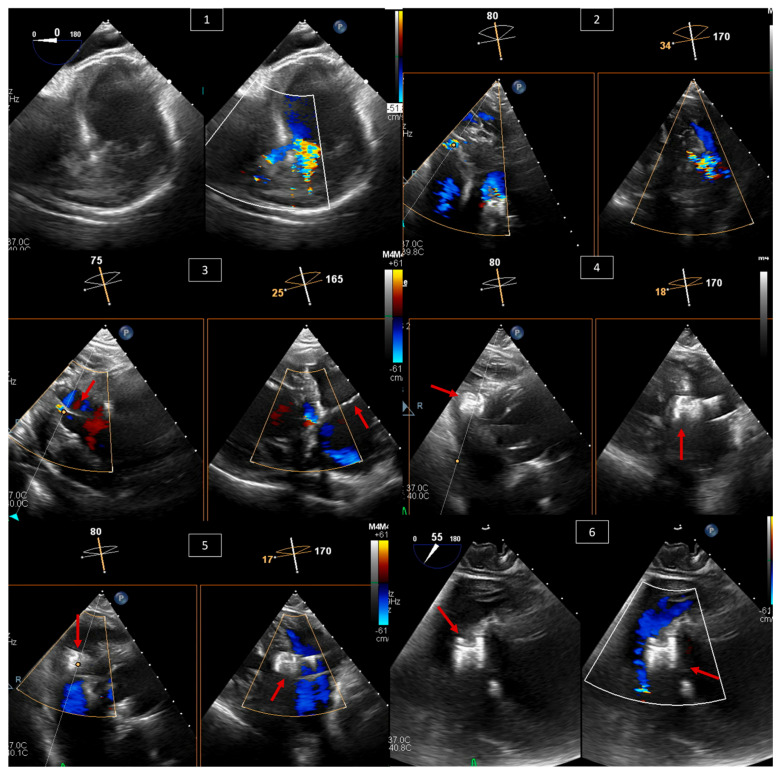
TEE of a percutaneous muscular VSD closure in a 54-year-old male with symptomatic significant left-to-right shunt. (**1**) Transgastric view of the muscular VSD with and without color Doppler showing left-to-right shunt. (**2**) X-plane on the defect with color Doppler at different angles to decide on the best angle for guiding the intervention. (**3**) X-plane of a transgastric view with color Doppler showing the wire (red arrow) across the VSD at 75 and 165 degrees. (**4**) X-plane of a transgastric view showing the VSD closure device (red arrow) in position and still attached to the wire at 80 and 170 degrees. (**5**) The same view from (**4**), now with color Doppler showing no residual left-to-right shunt. (**6**) Transgastric view at 55 degrees with color Doppler showing the VSD closure device (red arrow) after release in a stable position.

VSDs account for almost 20% of all ACHD defects [16,17]. This defect can occur anywhere along the ventricular septum. It ranges from small, restrictive, and asymptomatic, to large and significant, with symptoms of left heart failure due to left-to-right shunt and left ventricular overload. Indications for VSD closure are well discussed in the AHA/ACC as well as ESC guidelines [8,9], and these include evidence of left ventricular volume overload and a hemodynamically significant shunt (Qp:Qs ≥ 1.5:1) as long as pulmonary vascular resistance (PVR) is low. Patients with worsening aortic valve regurgitation or a history of infective endocarditis may also be candidates for VSD closure. Percutaneous closure of VSD is feasible for muscular or apical VSDs and some membranous VSDs [18,19]. Similar to ASD, imaging is crucial in percutaneous intervention. Periprocedurally, the echo should focus on characterizing the type of VSD, location relative to other structures, shunt direction, and sequelae of the shunt, and look for other congenital related defects. During the procedure, the echocardiographer should look for the best view to see the defect on TEE (transgastric view in Figure 5), and using it, guide the interventionalist in sizing, wire crossing, positioning the device, and evaluating device stability and interaction with nearby structures before and after deployment (Figure 5).

## 4. Transcatheter Edge-to-Edge Repair (TEER) of Systemic AV Valve

**Figure 6:**
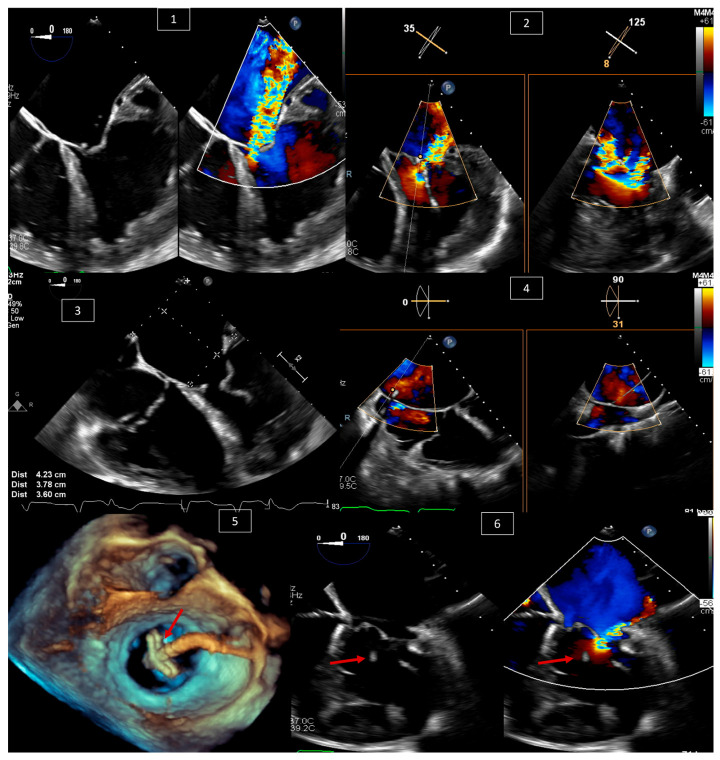
A 69-year-old male patient with a congenitally corrected transposition of the great arteries with severe tricuspid valve (systemic atrioventricular valve) regurgitation (TR). (**1**) Confirming the severity of the TR with and without color Doppler at 0 degrees and with X-plane color Doppler at 35 and 125 degrees in (**2**). (**3**) Measuring the size of the left atrium to confirm the feasibility of the clip procedure. (**4**) Guiding the crossing of the interatrial septum with X-plane color Doppler and confirming the position of the wire in the left atrium. (**5**) Three-dimensional view of the clipping device (red arrow) crossing the tricuspid valve from the left atrial view. (**6**) Looking at the tricuspid valve at 0 degrees after releasing the clip (red arrow), with and without color Doppler, and confirming the reduction in TR severity.

TEER is being widely studied and used for the treatment of mitral regurgitation in patients without congenital heart disease [20,21,22,23]. This use has expanded, and studies show the feasibility of the technique in surgical high-risk patients with congenital heart disease who have a systemic atrioventricular valve (AVV) other than the classical mitral valve, and develop severe systemic atrioventricular valve regurgitation. Such patients include those with congenitally corrected transposition of the great arteries (ccTGA), transposition of the great arteries with surgical atrial switch and systemic tricuspid valve regurgitation, or functional single ventricles with systemic AVV regurgitation [24,25,26]. Indications for intervention are beyond the scope of this article. The importance of TEE is to confirm the indication for and feasibility of the procedure by measuring the size of the atria, guiding the interatrial puncture when needed, and guiding the clipping device on the intended leaflets and its interaction with nearby structures (Figure 6).

## 5. Paravalvular Leak Intervention

**Figure 7:**
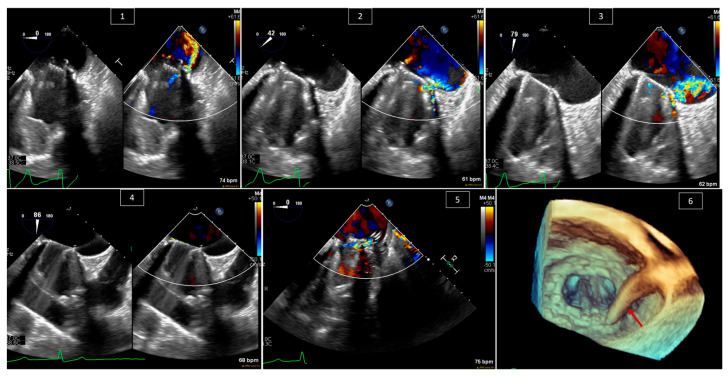
Paravalve leak plugging in a 51-year-old male with symptomatic paravalve leak. (**1**–**3**) Looking at the posterior paravalve area and the leak at different angles, with and without color Doppler—0 degrees in (**1**), 42 degrees in (**2**), and 79 degrees in (**3**). The intervention. (**4**) Guiding the wire across the paravalve leak and confirming its position with and without color Doppler. (**5**) Seeing the catheter inside the paravalve leak area with color Doppler. (**6**) The catheter (red arrow) in the paravalve leak area in 3D.

Many adult patients with congenital heart disease undergo valve replacement surgeries during their lives. One of the complications of such surgeries is paravalvular leak. Paravalvular leak is not uncommon; it has been reported in up to 18% of patients in the aortic valve position and in 23% in the mitral valve position [27,28]. Patients who have an indication for reintervention, such as severe symptomatic paravalvular regurgitation or hemolysis associated with paravalvular regurgitation [29,30], nowadays have the option of surgical or percutaneous intervention, with the latter being a lower-risk procedure that has been shown to be effective [31,32]. These procedures necessitate the guidance of TEE to guide the catheter and device into the paravalvular area and decide the size of the device to be used, as well as to check the final result and residual leaks (Figure 7).

## 6. Baffle Complications in Atrial Switch Operations

**Figure 8:**
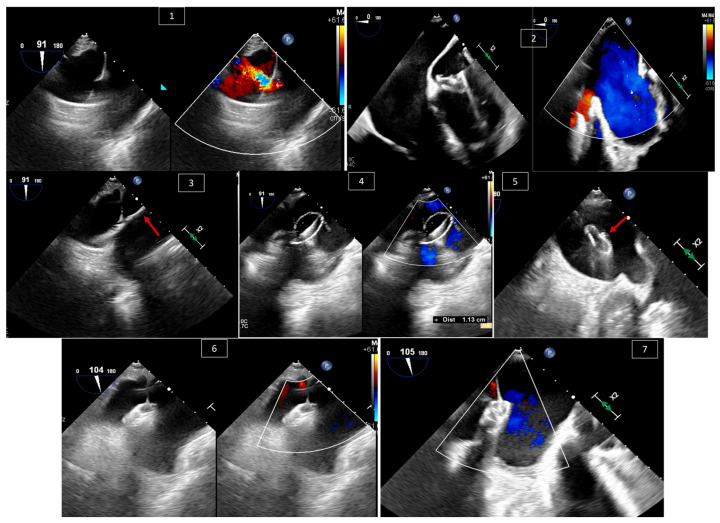
Baffle leak occlusion in a 46-year-old male patient with D-transposition of the great arteries post Mustard atrial switch operation and with a significant inferior systemic baffle leak. (**1**) Mid esophageal view at 90 degrees showing the systemic baffle leak with and without color Doppler, with flow crossing from the pulmonary venous baffle towards the IVC baffle. (**2**) Mid esophageal view at 0 degrees showing widely patent pulmonary venous baffle—easily recognized from the tricuspid valve (systemic AV-valve) with and without color Doppler. (**3**) Showing wire (arrow) across the systemic baffle leak. (**4**) Balloon sizing (1.13 cm) and occlusion of the defect with and without color Doppler. (**5**) Deploying the pulmonary venous baffle disc (arrow). (**6**) Deployment of the systemic venous baffle disc. (**7**) Final result with the device in a stable position and no more leaks visible by color Doppler.

Patients with dextrotransposition of the great arteries who underwent Mustard or Senning atrial switch operations are prone to baffle complications. These complications can be related to obstruction or leak of the baffle. Obstruction in the superior systemic baffle is more common than the inferior baffle, especially in the presence of a transvenous pacemaker or ICD leads in the baffle, and those are more common than obstruction in pulmonary venous baffles [33,34]. Treatment of these obstructions can be accomplished by ballooning and stenting stenosed areas using a transcatheter approach. On the other hand, baffle leaks complicate two thirds of atrial switch operations [35]. Baffle leaks may cause ventricular dilation, clinically significant desaturation at rest or with exercise, and increased risk for paradoxical embolization, and thus necessitate closure that can be accomplished using a transcatheter occlusion device. These procedures rely on TEE in guiding the ballooning and stenting of the stenosed baffle or the position of the occlusion device for baffle leaks. TEE is important in preventing complications in these procedures, such as impingement of a valve, erosion of devices, occlusion of a nearby baffle, etc. Figure 8 shows an example of systemic baffle leak occlusion.

## 7. Interventions in Fontan Circulation

**Figure 9:**
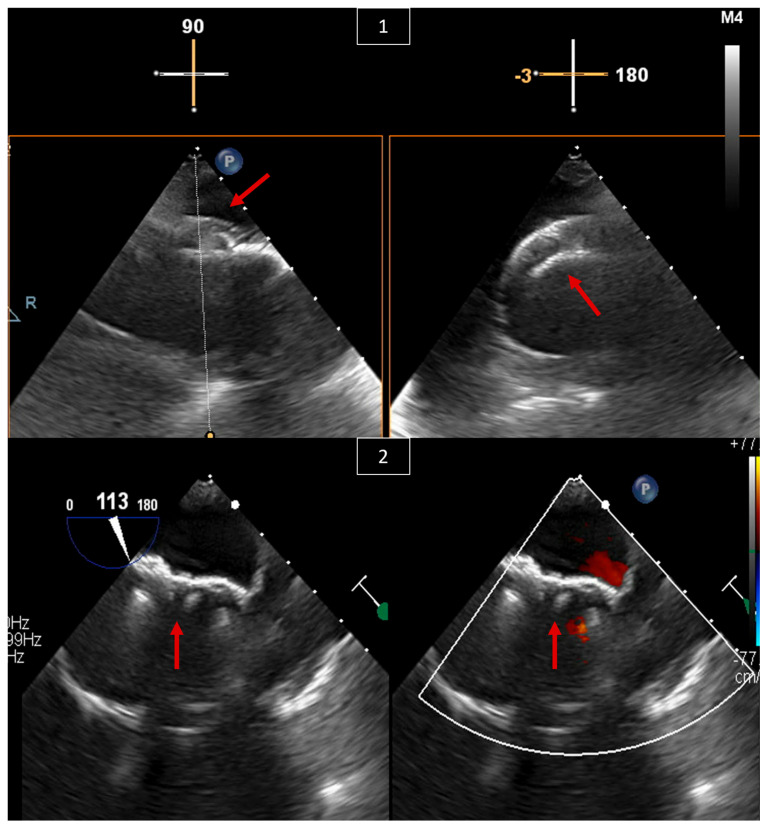
Fontan fenestration device closure in a 37-year-old male with lateral tunnel Fontan. (**1**) Mid esophageal view at 90 degrees with X-plane showing the fenestration closure device (red arrow) in stable position. (**2**) Showing the same device from (**1**) with and without color Doppler.

Univentricular heart physiology is rare; the prevalence is estimated to be 0.05–0.15 per 1000 live births [5,36]. With the advancement of medicine, these patients are able to survive to adulthood after undergoing multiple surgical procedures during their lifetimes, with Fontan circulation being the final stage for single ventricle palliation. Freedom from late complications of Fontan is uncommon; up to 50% of Fontan patients experience a complication related to Fontan circulation over a period of 20 years [37]. In order to prevent and treat these complications, these patients undergo frequent hemodynamic and interventional catheterizations. Some of the most common interventions include Fontan baffle ballooning and stenting, creation or closure of fenestrations (Figure 9), and trans-baffle puncture for electrophysiological interventions [38,39].

## 8. Other Interventions That Less Often Necessitate TEE During the Procedure

### 8.1. Transcatheter Pulmonary Valve Replacement (TCPVR)

Many patients with congenital heart defects undergo surgeries involving reconstruction of the right ventricular outflow tract (RVOT) with or without the use of conduits and valves. Later in life, these conduits and valves tend to dysfunction and need reintervention, surgically or percutaneously. The first percutaneous pulmonary valve replacement was first introduced in 2000 [40], and since then has gained much interest and popularity, becoming the most commonly performed transcatheter valve procedure in patients with ACHD [41,42,43,44]. Echocardiography has been used before these procedures in order to document the pathology, consequences, and indication for valve replacement. During the procedure, TEE has limited utility due to frequent difficulties in visualizing conduits and valves; this is where intracardiac echo (ICE) has been of significant advantage, and is used more often in these cases [42,45].

### 8.2. Coarctation of Aorta Ballooning/Stenting

Coarctation of the aorta is a localized stenosis or a hypoplastic segment of the aorta. The localized form is usually located near the region of the ductus arteriosus. The more diffused form is characterized by tubular hypoplasia and involves the distal aortic arch or the area distal to the origin of the left subclavian artery and the ductus area [46]. It is accountable for 2–5% of all congenital heart defects and has a 2:1 male predominance [5,47,48]. Transcatheter balloon angioplasty of aortic coarctation was initially performed in 1982 by Singer et al. [49], and since then the transcatheter-based procedure evolved to include stenting [50] and became the first-line therapy in most CoA cases [8,9]. Echocardiography plays a crucial rule in screening patients before the procedure, and in cases when radiation and contrast are of major concern, TEE can be used to guide the procedure and deployment of the stent [51].

### 8.3. Patent Ductus Arteriosus (PDA) Closure

PDA is an arterial communication between the proximal part of the descending aorta and the distal main pulmonary artery. It plays an important role in fetal life and usually closes spontaneously within the first few days of life. The prevalence of PDA in the general population is 0.29–0.87 per 1000 living births [6,36]. PDA originally produces left-to-right shunt. Small PDA with small left-to-right shunt (Qp:Qs < 1.5) are usually incidental findings. Medium and large shunt due to PDA (Qp:Qs > 1.5), if the patient reached adulthood, results in either left ventricular overload or pulmonary hypertension. Intervention is indicated when there are symptoms or significant left-to-right shunt with evidence of left volume overload and no evidence of significant pulmonary vascular resistance [8,9]. Percutaneous catheter closure of PDA has been performed for many years using different devices and coils [38,52]. The use of TEE during the procedure, although not necessary, can help in guidance, especially when there are concerns using contrast and radiation during the procedure [53,54].

## 9. Hybrid Procedures

Hybrid procedures refer to the relatively new approach to procedures when more than one subspecialty collaborates for the success of the procedure. In ACHD, a growing number of hybrid procedures have been described in which there was a collaboration between surgeons and interventionalists that made procedures doable and less invasive, and shortened procedure time, avoiding cardiopulmonary bypass, gaining vascular access, etc. In ACHD, these procedures include minimally invasive VSD occlusion, TCPVR in patients with large RVOTs, transcatheter mitral valve replacement in difficult anatomies, paravalvular leak occlusion through a transapical approach, and others [55,56,57,58,59]. These procedures may need the guidance of TEE, depending on procedure specifics.

## 10. The Future of Imaging During Congenital Heart Disease Procedures

Advancements in technology and the growing scope of percutaneous procedures in the congenital heart disease field underscore the critical need for parallel progress in imaging techniques. Intracardiac echocardiography (ICE) is one such technology experiencing expanded use in percutaneous interventions. This growth is driven not only by its excellent patient tolerance, reduced fluoroscopy exposure, and avoidance of general anesthesia or a second operator, but also by its unmatched ability to visualize anteriorly located structures, such as right ventricular outflow tract lesions, especially with the introduction of new ICE catheters [60].

Similarly, advancements in 3D live imaging are addressing the inherent limitations of 2D imaging, which struggles to comprehensively assess anatomical and morphological changes during procedures. Enhanced 3D echocardiographic imaging is increasingly being integrated into percutaneous interventions, offering faster, higher-quality visualization. These improvements enhance procedural feasibility and safety, significantly benefiting patient outcomes [61].

While these imaging technologies hold particular importance in the adult congenital heart disease (ACHD) field, a detailed discussion of them is beyond the scope of this article.

## 11. Conclusions

Transcatheter interventions in adult congenital heart disease (ACHD) are diverse and rapidly advancing. The majority of these procedures rely on transesophageal echocardiography (TEE) guidance, underscoring the critical role of an echocardiographer with extensive knowledge of both complex anatomy and procedural techniques. Such expertise is essential for ensuring procedural safety and success. This review highlights key considerations in some of the most commonly performed transcatheter interventions in ACHD. With ongoing technological advancements, the goal is to provide safer and more effective interventions, reducing the need for complex surgeries and improving patient outcomes.

## Data Availability

No new data were created or analyzed in this study. Data sharing is not applicable to this article.
